# A longitudinal study on determinants of the intention to start smoking among Non-smoking boys and girls of high and low socioeconomic status

**DOI:** 10.1186/s12889-015-1917-9

**Published:** 2015-07-13

**Authors:** Henricus-Paul Cremers, Liesbeth Mercken, Hein de Vries, Anke Oenema

**Affiliations:** Department of Health Promotion, School for Public Health and Primary Care (CAPHRI), Maastricht University, P.O. Box 616, 6200 MD, Maastricht, The Netherlands

**Keywords:** Socioeconomic status, Smoking intention, Primary school children, Gender differences, Socio-cognitive factors

## Abstract

**Background:**

This study identifies differences in socio-cognitive factors as they relate to the intention to smoke among boys and girls living in high socioeconomic status (HSES) and low socioeconomic status (LSES) neighborhoods.

**Methods:**

A total of 1,643 children (aged 10–12 years) completed a web-based questionnaire assessing their intention, attitude, social influences, and self-efficacy toward smoking at baseline and at one year follow-up. Logistic regression analyses were conducted to examine the relations between intention and predictor variables (i.e. attitude, social influence, and self-efficacy). Three-way interaction terms were added to the first analysis to examine potential interactions of gender, socioeconomic status and predictor variables. A 3-way interaction effect was present, and therefore subgroup analyses for HSES and LSES boys and girls were warranted.

**Results:**

The results indicated that positive attitudes toward smoking were related to the intention to smoke among HSES boys, whereas HSES girls had higher intentions to smoke if they perceived fewer disadvantages of smoking (OR: 0.42; 95 % CI: 0.22–0.82). The intention to smoke among LSES boys was predicted by perceived social norms (OR: 0.49; 95 % CI: 0.25–0.93); in LSES girls, the smoking behavior of people in their environment was most strongly related to their smoking intention (OR: 5.55; 95 % CI: 2.81–10.93).

**Conclusions:**

To prevent youth smoking, HSES boys and girls may benefit from interventions that address attitudes. Boys from an LSES neighborhood may profit from smoking prevention interventions that target social norms, while LSES girls may benefit from strategies aimed at resisting the influence of smokers in their environment.

**Trial registration:**

The ‘Fun without Smokes’ study is approved by the Medical Ethics Committee of the Atrium-Orbis-Zuyd Hospital (NL32093.096.11/MEC 11-T-25) and registered in the Dutch Trial Register (NTR3116).

## Background

After children transition from primary to secondary school, the prevalence of smoking increases rapidly [1]. The reasons for the increase in smoking prevalence includes periods of high temptation for engaging in unhealthy behaviors (i.e. smoking) [2, 3] and children’s aims of belonging to specific groups. Smoking prevention programs may be effective to prevent smoking among this age group [4, 5]. However, before such programs are developed, it is crucial to identify underlying determinants of children’s smoking intention or behavior, in order to target the right determinants for the right children. Several determinants of smoking intention and behavior have been identified among young people in previous studies (e.g. perceived smoking behavior of people in their environment, positive attitudes toward smoking, and pubertal development) [6–11]. To date, there is no conclusive evidence as to whether the same determinants are important for all children, or whether there are differences between boys and girls of a lower or higher socioeconomic status (SES). Insight into factors that are associated with the intention to smoke in those subgroups may increase the opportunity to better tailor smoking prevention programs to these specific groups and may make such programs more effective.

SES has been correlated with children’s smoking initiation [7] and the intention to start smoking [12]. Low socioeconomic status (LSES) adolescents, for example, engage in smoking more often than high socioeconomic status (HSES) adolescents [6, 10, 13]. Moreover, prior research indicates that socio-cognitive factors (such as attitude, perceived social influence, and self-efficacy expectations) may be involved in adolescents’ decision to start smoking [6]. Even though young people express negative attitudes toward smoking [14, 15], previous studies have shown that LSES youngsters had a more positive attitude toward smoking, were less able to refuse cigarettes, were more encouraged by their social environment to start smoking, and had more smokers in their direct environment [6, 16–18] compared to HSES youngsters.

Several studies indicate that there are also gender differences in cigarette smoking [19–21] and smoking initiation among youth [22], where smoking prevalence rates are higher among girls than boys [23, 24]. Girls may believe that smoking enables them to retain a slim body or reduce their weight [25], while boys may be more influenced to start smoking when they perceive fewer disadvantages to smoking [9]. Furthermore, girls engage in smoking more often due to the perceived influences of parents or friends [9, 26], while boys begin smoking more frequently when their peers are smokers [27]. However, there is some inconsistency in these findings, as other studies have reported that parental or friends’ smoking behavior predicted the onset of smoking in both boys and girls [28, 29].

Differences in socio-cognitive factors have been separately observed for SES and gender concerning adolescents’ smoking behavior. No studies were found to have investigated influential factors concerning the intention to start smoking in HSES boys, HSES girls, LSES boys and LSES girls. Therefore this study aims to examine the influence of socio-cognitive factors (i.e. attitude, social influence and self-efficacy expectations) on children’s intention to start smoking by examining the differences between boys and girls in an HSES environment and boys and girls in an LSES environment.

## Methods

### Design, participants, & procedure

This study had a longitudinal design and used baseline (T0) and one year follow-up (T1) data from a smoking prevention intervention trial called “Fun without Smokes” [30]. The “Fun without Smokes” study is a web-based, computer-tailored smoking prevention intervention that was evaluated in a cluster-randomized controlled trial. At T0 (October 2011) and T1 (October 2012), children completed a web-based questionnaire concerning their smoking behavior, intention, attitude, social influence, and self-efficacy expectations toward (non-) smoking. After completion of the baseline assessment, children in the experimental group received computer-tailored feedback letters via email and at the “Fun without Smokes” website. Children in the control group did not receive feedback letters. A detailed description of the “Fun without Smokes” intervention study is available elsewhere [30].

Children in the “Fun without Smokes” study were recruited through primary schools by Municipal Health Promotion Organizations and Maastricht University. Children in grade 7 (aged 10–11 years) were eligible to participate in the intervention study. Approximately 3,500 Dutch primary schools were approached for participation in the smoking prevention study, but only 162 primary schools decided to participate (*N* = 3,213 children). In the present study a passive informed consent procedure was used in which all children of the participating schools received informed consent letters for their parents or guardians. If children, parents, or guardians refused to be involved in the “Fun without Smokes” study they were able to sign the informed consent letter and return it to the children’s teacher (1.7 % refused). Subsequently, the teachers informed the research team about the children that refused participation. At T1, 2,146 children (33.2 % drop-out rate) from 133 primary schools filled out the follow-up measurement with the same group of children now in grade 8 (aged 11–12 years). Both baseline and follow-up measurements were completed in the classroom under teacher supervision. In the present study, only the responses from children who had completed both measurements and had provided a verifiable postal code were included in the analyses.

After one year of follow-up, no intervention effects were observed; therefore, it was possible to use the longitudinal data of “Fun without Smokes” for the present study.

### Measurements

The primary outcome measure was intention to smoke. The intention to smoke was assessed by self-reports using a previously used question [31, 32]: “Do you intend to start smoking in the future?” A five-point Likert scale was used in the answer format (1 = certainly yes; 2 = yes; 3 = I don’t know; 4 = no; 5 = certainly not). Children who indicated ‘no’ or ‘certainly not’ were categorized as not having the intention to smoke (coded as 0). Otherwise, children were categorized as having the intention to smoke (coded as 1). It was expected that children who were undecided about smoking were more inclined to engage in smoking compared to children who were certain about not smoking. For that reason, children who indicated ‘I don’t know’ were also categorized as having the intention to smoke.

SES of the participating children was based on the SES index score of the areas in which they live, as determined by their postal code. All postal codes have an SES index score. This SES index score was retrieved from the Netherlands Institute for Social Research (a Dutch government agency that conducts research into the social aspects of all areas of government policy), which gathers information from all Dutch inhabitants concerning their income, occupation, and education. These indicators were used to calculate an SES index score for the 4-digit postal code areas. Thus, SES index scores indicated social status at a neighborhood level [33, 34]. The SES index score ranges from +3.4 to–5.2 and is based on Dutch inhabitants’ income, occupation, and education. All scores higher than zero were indicated as high SES. The higher this SES index score, the higher the SES of the child. SES index lower than or equal to zero indicated low SES. The lower the SES index score, the lower the SES of the child. In the present study, children from an LSES neighborhood were coded with a ‘0’ and children from an HSES neighborhood were coded with a ‘1’.

Background variables included the age (in years), gender (1 = boy; 2 = girl) and ethnicity of the participating children. In line with the guidelines of Statistics Netherlands, a child was deemed to have a Western ethnic background (scored as 1) if he/she and both parents had been born in the Netherlands, another European country, North America, Oceania, Indonesia (a former Dutch colony), or Japan. Otherwise the child was deemed to have a non-Western ethnic background (scored as 2) [35].

The socio-cognitive constructs were derived from the integrated model for exploring motivation and behavioral change (I-Change model) [36].

The attitude dimension advantage was measured by assessing the positively perceived consequences of smoking using nine items. Participants answered these questions by using their perception of the various benefits of smoking, such as feeling more mature, sociable, cool, or receiving more attention from friends. Children were asked to complete the following question “If I smoke….” with a four-point answer category ranging (for example) from 4 = ‘I will feel very mature’ to 1 = ‘I will not feel mature’ (Cronbach’s alpha = 0.84).

The attitude dimension disadvantage was measured using ten different negatively perceived consequences of smoking, such as I will become less physically fit, I will become ill or, I will become addicted. Children provided an answer on a four-point scale ranging (for example) from 4 = ‘I will become very ill’ to 1 = ‘I will not become ill’ (Cronbach’s alpha = 0.80).

Social norm was assessed through the perceptions of smoking norms of important people in the child’s environment. Children had to complete seven questions addressing their father, mother, brother (s), sister (s), friends, best friend, and most people important to them. For example, “My mother thinks that I….”. These questions could be scored on a five-point Likert scale ranging from +2 = ‘definitely should not smoke’ to -2 = ‘definitely should smoke’ (Cronbach’s alpha = 0.69).

Modeling was measured by assessing the smoking behavior of parents, siblings, family, and friends. A total of eight questions were asked, such as: “Does your mother/father/brother (s)/sister (s)/best friend smoke?” (the five-point answer formats ranged from 5 = ‘often’ to 1 = ‘never’) and “How many of your friends/other family members/classmates smoke?” (the five-point answer scales ranged from 5 = ‘(almost) all’ to 1 = ‘(almost) none’). Children could also indicate that they had no parents, siblings, family, or friends or that they did not know if people in their social environment smoked; these answers were also categorized as ‘1’. To create a single variable for modeling, an average score was calculated: the scores of all individual items (best friend, mother, father, brother (s), sister (s), friends, other family members, and classmates) were added and divided by the number of questions. Therefore, a higher score on this scale indicated that more people smoked in the child’s social environment.

Self-efficacy expectations were measured with ten questions to assess the child’s ability to refuse cigarettes in different situations. For example, the question was posed “When others smoke it is….for me not to smoke”; this question was answered using a five-point Likert scale ranging from +2 = ‘very easy’ to -2 = ‘very difficult’ (Cronbach’s alpha = 0.93).

### Analyses

Descriptive analyses (means and percentages) were performed to describe the sample under study. This sample included only non-smoking children. Potential differences between boys and girls of HSES and LSES neighborhoods were assessed through an analysis of variance (ANOVA) using Gabriel’s pairwise comparison test (GABRIEL). This post-hoc test is suitable for unequal sample sizes [37] and adjusts for multiple testing. Significant differences observed between the subgroups indicate that they potentially modify the effects of the socio-cognitive factors. Therefore, factors that differed significantly between subgroups were included as interaction terms (with attitude, social influence and self-efficacy) in the analyses to test for effect modification. If those interaction terms were found to be significant, they were included in the main analyses regarding the subgroups. The influence of school and class level on the smoking intention of participating children was analyzed to test for possible nesting effects. The variance of the random intercept of both the school and class level was zero, indicating that multilevel analyses were not warranted.

To identify whether SES and gender moderated the association of predictor variables (i.e. attitude, social influence and self-efficacy expectations) with the outcomes 3-way interaction terms (SES by gender by predictor) were included in a logistic regression analysis. The analyses were adjusted for age and ethnicity. If a 3-way interaction effect was determined to be present, subgroup analyses were carried out for boys and girls living in HSES or LSES environments. All analyses were performed in SPSS 20.0. The significance level was set at *p* ≤ 0.05. To reduce potential type I errors, the interaction effects were considered significant if the *p*-value was equal to or lower than 0.10.

## Results

### Basic characteristics

A total of 1,643 children met the inclusion criteria (76.6 %) and were included in the analyses. The mean age was 11.35 years and most had a Western ethnic background (90.1 %). At T0, 13.5 % of the children indicated to have the intention to start smoking (9.1 % indicated ‘I don’t know’) and at T1 this was 11.6 % (7.9 % indicated ‘I don’t know’). As shown in Table 1, 376 boys and 470 girls from an HSES neighborhood participated, whereas 348 boys and 449 girls from an LSES environment were included in the analyses. Boys from an HSES environment had a Western ethnic background significantly more often than girls from an HSES environment (*p* = 0.02). However, ethnicity was not an effect modifier in the present study (*p* > 0.10).

**Table 1:** Basic characteristics of the total sample and subgroups based on gender and SES

	Total sample (*N* = 1,643)	HSES boys^A^ (*N* = 376)	HSES girls^B^ (*N* = 470)	LSES boys^C^ (*N* = 348)	LSES girls^D^ (*N* = 449)	P*
Age (in years)	11.35	11.38	11.30	11.37	11.35	
Ethnicity (% Western)	90.1	93.1	87.0	91.7	89.8	A > B
Intention to smoke at T0 (% yes)	13.5	13.8	14.3	15.8	10.7	
Intention to smoke at T1 (% yes)	11.6	11.7	9.4	14.9	11.1	

*Significant at 0.05 level

Note: ^A^boys living in an HSES environment; ^B^girls living in an HSES environment; ^C^boys living in an LSES environment; ^D^girls living in an LSES environment

### Interaction effects with SES and gender

The 3-way interaction term of SES by gender by modeling was significant (OR: 0.19; 95 % CI: 0.04–1.00). For that reason, separate analyses were performed for boys and girls of an HSES environment and boys and girls of an LSES environment.

### Results of analyses stratified by SES and gender

The results of the stratified analyses are presented in Tables 2 and 3. The intention to smoke for boys of an HSES neighborhood was especially predicted by the perceived advantages (OR: 2.39; 95 % CI: 1.27–4.49) and perceived low disadvantages of smoking (OR: 0.52; 95 % CI: 0.27–0.99) at T0. The intention to start smoking among girls living in an HSES environment were predicted by low perceived disadvantages (OR: 0.42; 95 % CI: 0.22–0.82) of smoking and the intention to engage in smoking at T0 (OR: 4.38; 95 % CI: 1.89–10.15).

**Table 2:** Association between socio-cognitive factors and intention at T1 between boys and girls of an HSES neighborhood

	HSES boys^A^			HSES girls^B^		
	OR^C^	95 % CI^D^	*P*	OR^C^	95 % CI^D^	*P*
Attitude (advantages)	**2.39**	**1.27 – 4.49**	**<0.01**	1.16	0.63 – 2.14	0.63
Attitude (disadvantages)	**0.52**	**0.27 – 0.99**	**0.05**	**0.42**	**0.22 – 0.82**	**0.01**
Social norm	0.69	0.32 – 1.50	0.35	0.89	0.40 – 1.98	0.78
Modeling	2.18	0.88 – 5.38	0.09	1.00	0.36 – 2.76	0.99
Self-efficacy	1.01	0.65 – 1.57	0.97	0.71	0.45 – 1.11	0.13
Age	0.72	0.37 – 1.39	0.32	1.56	0.80 – 3.02	0.19
Ethnicity	0.36	0.04 – 3.06	0.35	0.71	0.22 – 2.23	0.55
Intention to smoke at T0	2.40	0.97 – 5.92	0.06	**4.38**	**1.89 – 10.15**	**<0.01**

Note: ^A^boys living in an HSES environment; ^B^girls living in an HSES environment; ^C^Odds ratio; ^D^Confidence interval

**Table 3:** Association between socio-cognitive factors and intention at T1 between boys and girls of an LSES neighborhood

	LSES boys^A^			LSES girls^B^		
	OR^C^	95 % CI^D^	*P*	OR^C^	95 % CI^D^	*P*
Attitude (advantages)	1.32	0.67 – 2.61	0.43	0.98	0.50 – 1.94	0.96
Attitude (disadvantages)	0.76	0.43 – 1.33	0.33	0.53	0.26 – 1.06	0.07
Social norm	**0.49**	**0.25 – 0.93**	**0.03**	0.91	0.39 – 2.15	0.84
Modeling	1.87	0.84 – 4.15	0.12	**5.55**	**2.81 – 10.93**	**<0.01**
Self-efficacy	0.69	0.46 – 1.03	0.07	0.86	0.53 – 1.39	0.54
Age	1.42	0.77 – 2.61	0.26	1.05	0.57 – 1.93	0.88
Ethnicity	0.39	0.09 – 1.60	0.19	0.32	0.07 – 1.39	0.13
Intention to smoke at T0	2.10	0.92 – 4.81	0.08	**3.94**	**1.57 – 9.85**	**<0.01**

Note: ^A^boys living in an LSES environment; ^B^girls living in an LSES environment; ^C^Odds ratio; ^D^Confidence interval

For boys living in an LSES environment the perceived social norm at T0 (OR: 0.49; 95 % CI: 0.25–0.93) best predicted their intention to start smoking at T1. Intention to smoke among girls living in an LSES neighborhood was best predicted by the baseline perception of the smoking behavior of the people in their environment (i.e. modeling) (OR: 5.55; 95 % CI: 2.81–10.93) and their intention to start smoking at T0 (OR: 3.94; 95 % CI: 1.57–9.85) (Table 3).

## Discussion

The purpose of the present study was to examine the influence of socio-cognitive factors (i.e. attitude, social influence, and self-efficacy expectations) on children’s intention to start smoking by investigating the differences between boys and girls living in an HSES environment and boys and girls living in an LSES environment. Stratified analyses indicated that the intention to engage in smoking among boys and girls from an HSES neighborhood was predicted by more positive and less negative opinions toward smoking; social norms and modeling were respectively the most important factors predicting the intention to smoke for boys and girls living in an LSES environment. For girls in both an HSES and LSES neighborhood, the intention to start smoking at baseline predicted their intention to start smoking during the follow-up after 12 months.

In line with our findings, a study by de Vries [6] reported that smoking among LSES youngsters seemed to be nested in their social culture (i.e. receiving more influence from smokers in their environment, perceiving a higher social norm, or feeling more pressure to smoke), whereas HSES youngsters often linked smoking with more potential advantages (i.e. discovery of taste or reducing boredom) and disadvantages (i.e. unpleasant smell or risk of cancer). This study, however, was performed among Dutch adolescents (aged 12 – 16 year) and did not include children. The finding that social influence is significant among children of LSES neighborhoods is supported by previous studies regarding smoking behavior of adolescents [17, 18] and the intention to start smoking among children [12]. Due to the higher number of smokers in LSES neighborhoods [38], LSES children have greater access to cigarettes and are more frequently exposed to smokers [39]. Previous research also indicated that LSES parents have fewer or no smoking bans [40], which may increase the likelihood that children of LSES neighborhoods perceive smoking to be the norm. The present study indicates that different factors are influential in producing a higher intention to start smoking among boys and girls in an LSES environment (through social norms and modeling, respectively). Previous research demonstrated that girls seemed to be more susceptible to social influences [41–43]. A study by Hampson et al. indicated that boys may be more likely to have a higher intention to smoke due to the perceived social norms of their peers [15], which may be related to the hostile behavior of boys. However, these latter studies only focused on gender differences and did not investigate SES differences between boys and girls. Although differences between boys and girls of LSES neighborhoods were observed in the present study, prior studies [9, 44] reported inconsistent findings regarding perceived social norms or modeling among boys and girls. Therefore, more research is required to replicate those unique findings. The present study indicates another finding: positive and negative attitudes were most influential in producing higher intentions to start smoking among boys and girls from HSES neighborhoods. Perhaps children of HSES neighborhoods perceive other opinions and beliefs toward smoking because they are less exposed to the smoking behavior of others. Furthermore, this work reported that only the intention to start smoking at baseline among girls of LSES and HSES environments explains their smoking intention during the follow-up. This may be because girls engage in smoking more often than boys in the Netherlands, though boys catch up at a later age [1]. Girls may develop stronger intentions to engage in smoking at a younger age. Although it is hard to explain the differences between boys and girls from an HSES environment, it is advisable that future computer-tailored interventions take them into account. Another finding of the present study is that the intention to start smoking decreased after children participated 12 months in the “Fun without Smokes” study, whereas an increase in intention to start smoking is expected at an older age. This inconsistency may be explained since children’s knowledge and awareness levels were raised by only filling-out the web-based questionnaire at T0 and T1, which may have changed their opinion towards smoking or the intention to engage in smoking.

The present study indicated that to prevent the onset of youth smoking different socio-cognitive factors must be targeted to influence children’s intention to start smoking for both boys and girls in LSES and HSES environments. This information is important to refine the provision of feedback in computer-tailored programs. Through computer-tailored interventions, behavior change techniques can reach a large group of individuals and potentially improve their health related behaviors [45]. Those interventions promise to specify the personal characteristics (i.e. age, gender, SES) or the socio-cognitive factors (i.e. attitude, social influence or self-efficacy expectations) of an individual [46] to change their health related behaviors. Prior research indicates that computer-tailored interventions can prevent the uptake and continuation of smoking among children [31] and adolescents [47]. The results of the present study indicate that among boys of an HSES neighborhood the focus should be on modifying the perception of the advantages and disadvantages of smoking, whereas among girls of an HSES neighborhood the disadvantages toward smoking should be especially considered. Among girls in LSES neighborhoods the smoking behavior of people in their environment (modeling) should be addressed, while among boys in LSES neighborhoods the focus should be on the perceived social norm. Behavior change techniques can be incorporated into (computer-tailored) programs to change attitudes; these may include methods such as anticipated regret (focusing on feelings after children have smoked), repeated exposure (repeatedly showing negative consequences of smoking), arguments for not smoking [48], or providing information on the consequences of smoking [49]. Social norm and modeling may be influenced by various techniques such as providing information about others’ approval (providing information about what others think about the child’s smoking behavior) or providing opportunities for social comparison [48, 49].

### Strengths & limitations

The strengths of the present study include its large sample size and longitudinal design, which allowed for interpretation of the findings as causal relationships. There were also several limitations. First, due to several reasons (i.e. closed schools or schools that no longer wanted to cooperate) 29 schools refrained from participation at T1. Therefore, 33.2 % of the children that participated at T0 dropped-out after 12 months of follow-up. Drop-out may limit the findings of a study. However, it is expected that drop-out did not have much impact on the present study since a large sample size could still be included in the analyses and the drop-out rates were approximately equally distributed among the different subgroups. Second, there was no SES indicator at the individual level and so an SES indicator of the children’s neighborhood was used. This may imply that the SES score was not completely accurate for all of the children included in the study. However, the SES score of the neighborhood was based on the individuals living in that neighborhood. For that reason, an SES score of the neighborhood may highly correlate with an individual’s SES score [34]. Nevertheless, it is advisable that future research assess individual SES scores for participating children. A final limitation is that the stratified analyses were based on a single significant interaction term (SES by gender by modeling). Although, clear differences were observed regarding the influence of the smoking behavior of people in LSES and HSES neighborhoods, the findings of the other socio-cognitive factors (i.e. attitude and social norm) may be based on chance since no significant interaction terms were observed. However, the significant 3-way interaction indicated that there were differences between SES, gender and socio-cognitive factors concerning children’s intention to start smoking. Findings of the present study provide important implications for future research though additional studies are needed to support the results.

## Conclusion

Findings of the present study indicate that different socio-cognitive factors may be associated with the intention to smoke in boys and girls of HSES and LSES neighborhoods. The intention to start smoking for girls in an LSES environment was best predicted by the smoking behavior of people in their environment, whereas the intention to start smoking among boys of an LSES environment was most strongly associated with perceived social norms. Among boys and girls living in an HSES environment, the intention to start smoking was most strongly associated with more perceived advantages of smoking and less perceived disadvantages of smoking. For that reason, future smoking prevention programs among children of HSES neighborhoods may benefit from addressing the positive and negative consequences of (non-) smoking. Children of LSES neighborhoods may be better suited to strategies about resisting the smoking behaviors of people in their environment or strategies to cope with the perceived norms of influential people. Those differences in socio-cognitive factors may be incorporated in computer-tailored programs. It is, however, advisable for future research to first explore those possibilities among different subgroups.

## Abbreviations

HSES: High socioeconomic status
LSES: Low socioeconomic status
SES: Socioeconomic status
OR: Odds Ratio
CI: Confidence Interval
ANOVA: Analysis of variance
GABRIEL: Gabriel’s pairwise comparison test
SPSS: Statistical Package for the Social Sciences.
